# Supplementary material for: Minimizing Reference Bias with an Impute-First Approach
Source: bioRxiv. 2024 May 16:2023.11.30.568362. Originally published 2023 Dec 2. Preprint. [Version 2] doi: 10.1101/2023.11.30.568362 (PMC10705441; doi:10.1101/2023.11.30.568362)
Supplement: Supplement 1 [file NIHPP2023.11.30.568362v2-supplement-1.pdf]

bioRxiv preprint doi: https://doi.org/10.1101/2023.11.30.568362; this version posted May 16, 2024. The copyright holder for this preprint (which was not certified by peer review) is the author/funder, who has granted bioRxiv a license to display the preprint in perpetuity. It is made available under aCC-BY 4.0 International license.

Supplementary Figures

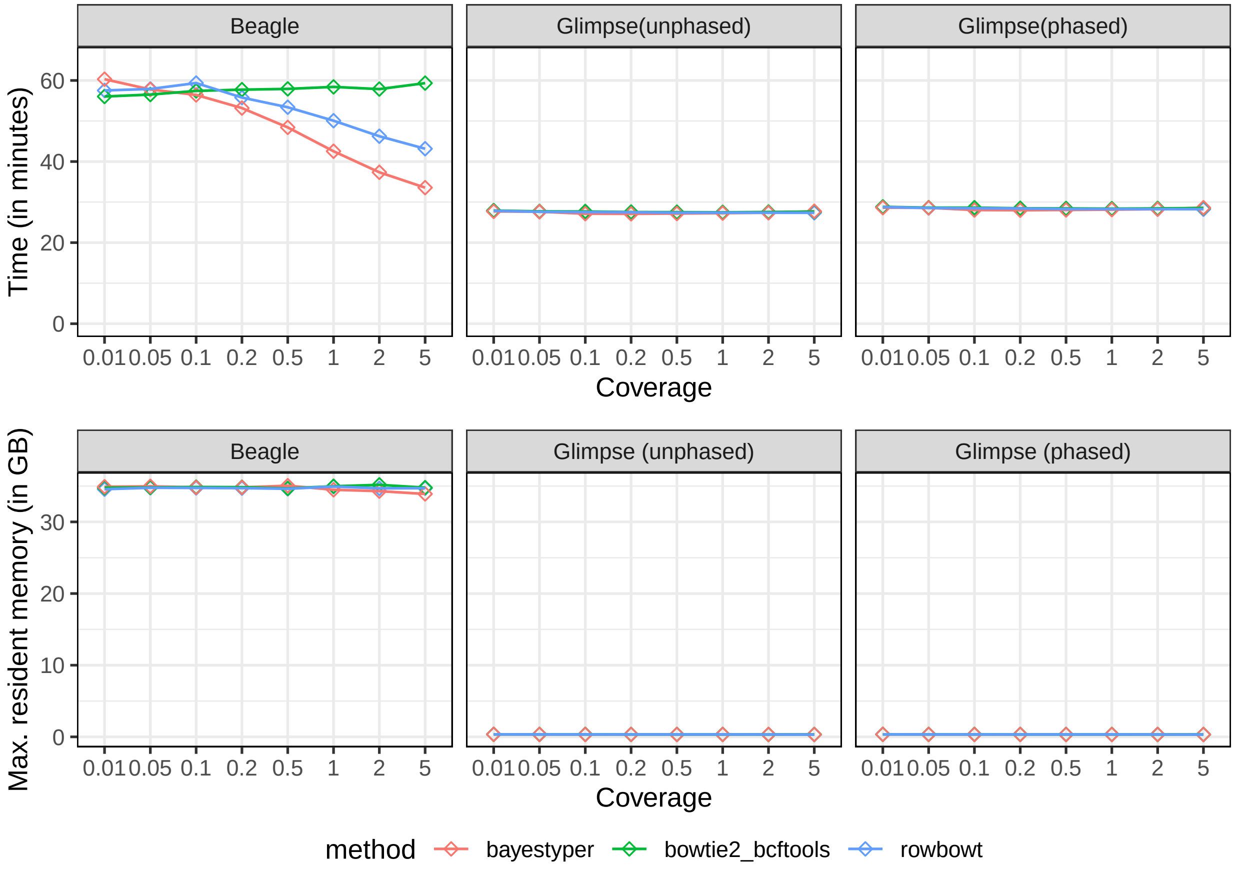

Figure S1: Computational overhead for Beagle and Glimpse stratified by the genotyping tool used upstraem, and as a function of the read coverage provided to the genotyper. Measurements for Glimpse were taken in both its phased and unphased modes.

bioRxiv preprint doi: https://doi.org/10.1101/2023.11.30.568362; this version posted May 16, 2024. The copyright holder for this preprint (which was not certified by peer review) is the author/funder, who has granted bioRxiv a license to display the preprint in perpetuity. It is made available under aCC-BY 4.0 International license.

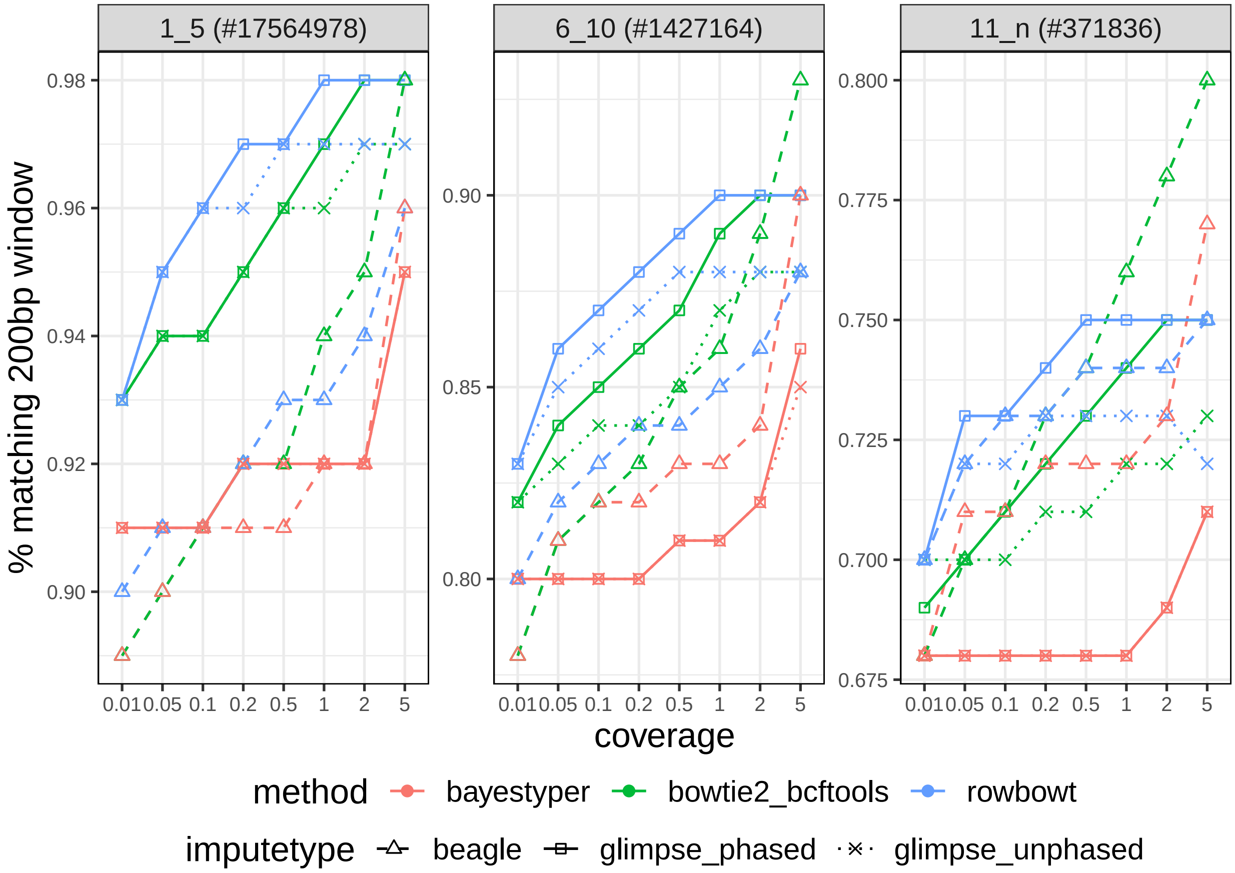

Figure S2: Window Accuracy for Beagle and Glimpse (in both phased and unphased modes) for different methods on diverse coverages.

bioRxiv preprint doi: https://doi.org/10.1101/2023.11.30.568362; this version posted May 16, 2024. The copyright holder for this preprint (which was not certified by peer review) is the author/funder, who has granted bioRxiv a license to display the preprint in perpetuity. It is made available under aCC-BY 4.0 International license.

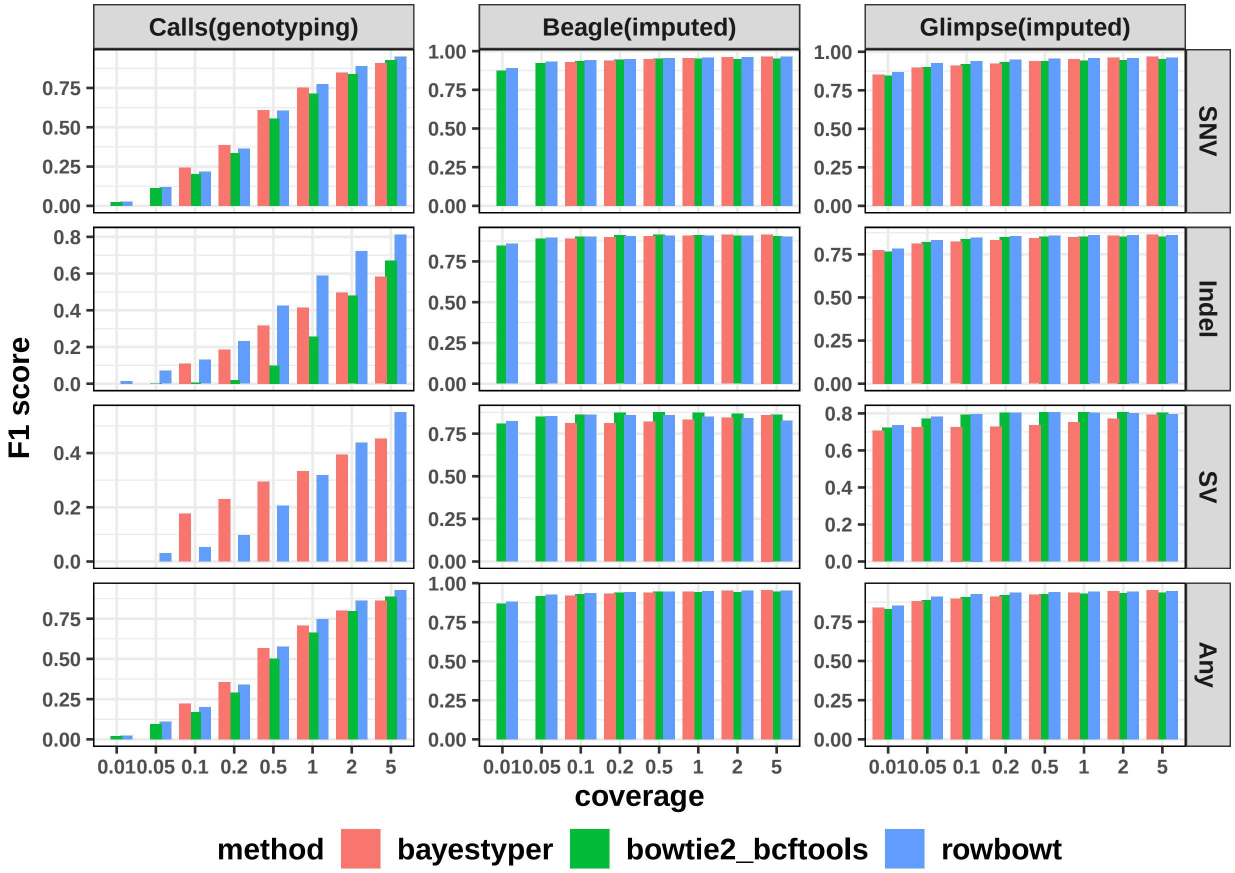

Figure S3: F1 score of alternate allele calls (ALT), stratified by variant type, for the personalized genomes constructed using each alignment/genotyping method in the Impute-first alignment workflow.

bioRxiv preprint doi: https://doi.org/10.1101/2023.11.30.568362; this version posted May 16, 2024. The copyright holder for this preprint (which was not certified by peer review) is the author/funder, who has granted bioRxiv a license to display the preprint in perpetuity. It is made available under aCC-BY 4.0 International license.

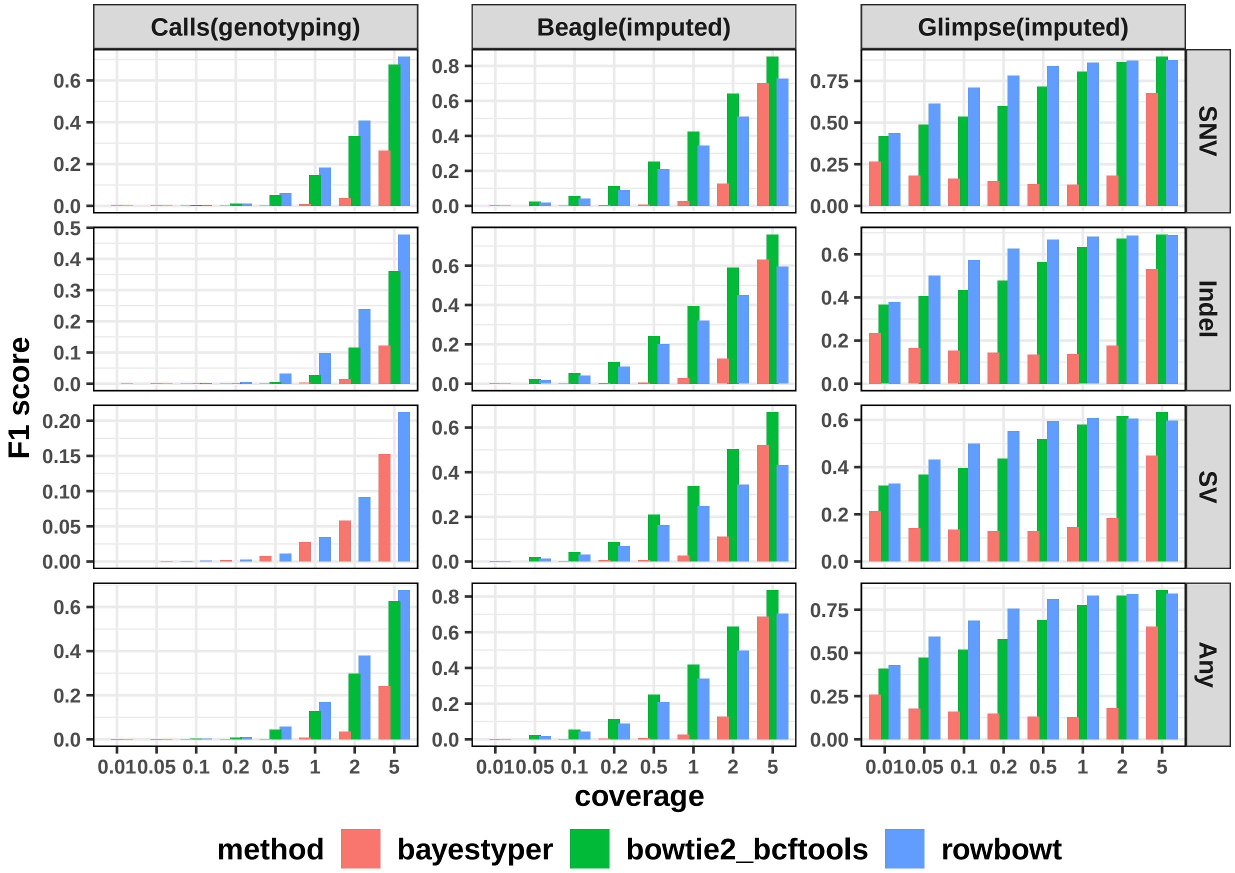

Figure S4: F1 score of heterozygous calls (HET), stratified by variant type, for the personalized genomes constructed using each alignment/genotyping method in the Impute-first alignment workflow.

bioRxiv preprint doi: https://doi.org/10.1101/2023.11.30.568362; this version posted May 16, 2024. The copyright holder for this preprint (which was not certified by peer review) is the author/funder, who has granted bioRxiv a license to display the preprint in perpetuity. It is made available under aCC-BY 4.0 International license.

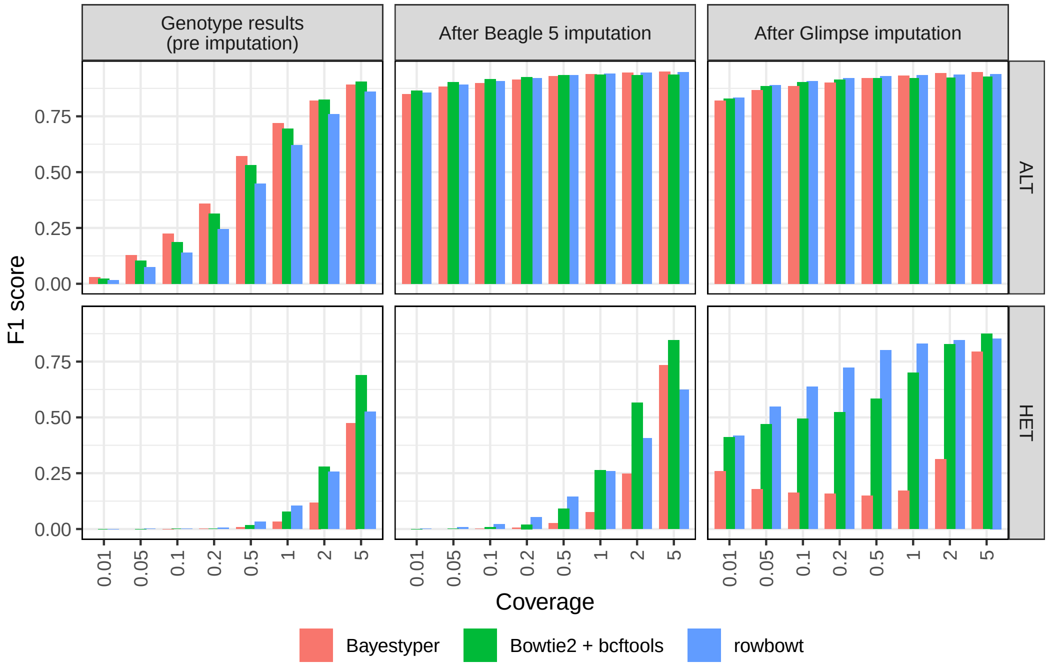

Figure S5: Aggregate F1 scores of alternate allele calls (ALT) and heterozygous calls (HET) across all variant types, generated using each alignment/genotyping method in the Impute-first alignment workflow on HG002.

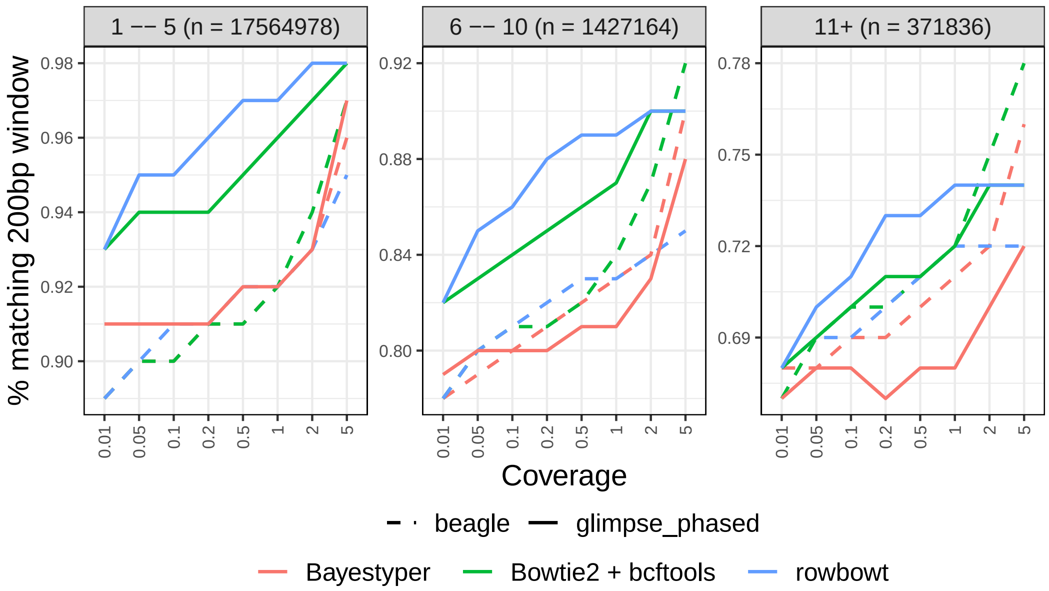

Figure S6: Window accuracy for diploid personalized genomes using HG002 data. The imputed sequence for each 200-bp windows anchored to a polymorphic site was compared to truth NA24385 sequence. Results are stratified by the number of polymorphic sites in the window.

bioRxiv preprint doi: https://doi.org/10.1101/2023.11.30.568362; this version posted May 16, 2024. The copyright holder for this preprint (which was not certified by peer review) is the author/funder, who has granted bioRxiv a license to display the preprint in perpetuity. It is made available under aCC-BY 4.0 International license.

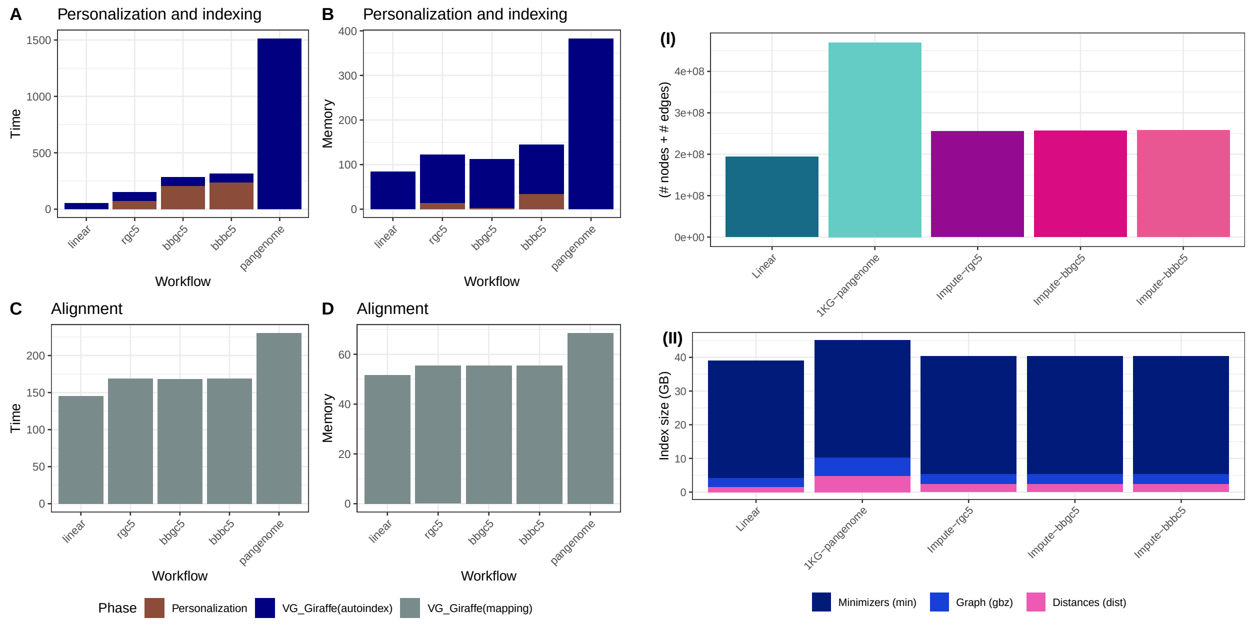

(a) Time and Memory Overhead of workflows within VG Giraffe framework.

(b) Graph and index sizes of workflows generated within VG Giraffe framework.

Figure S7: Computational efficiency of VG Giraffe-based workflows on HG002, considering time, memory footprint, and index size. Compared to typical pangenome VCF usage in VG Giraffe, the personalized VCFs used in Impute-first pipelines (*rgc5*, *bbgc5*, *bbbc5*) offered more efficient reference representation and were also comparable to the linear method in terms of computational efficiency.

bioRxiv preprint doi: https://doi.org/10.1101/2023.11.30.568362; this version posted May 16, 2024. The copyright holder for this preprint (which was not certified by peer review) is the author/funder, who has granted bioRxiv a license to display the preprint in perpetuity. It is made available under aCC-BY 4.0 International license.

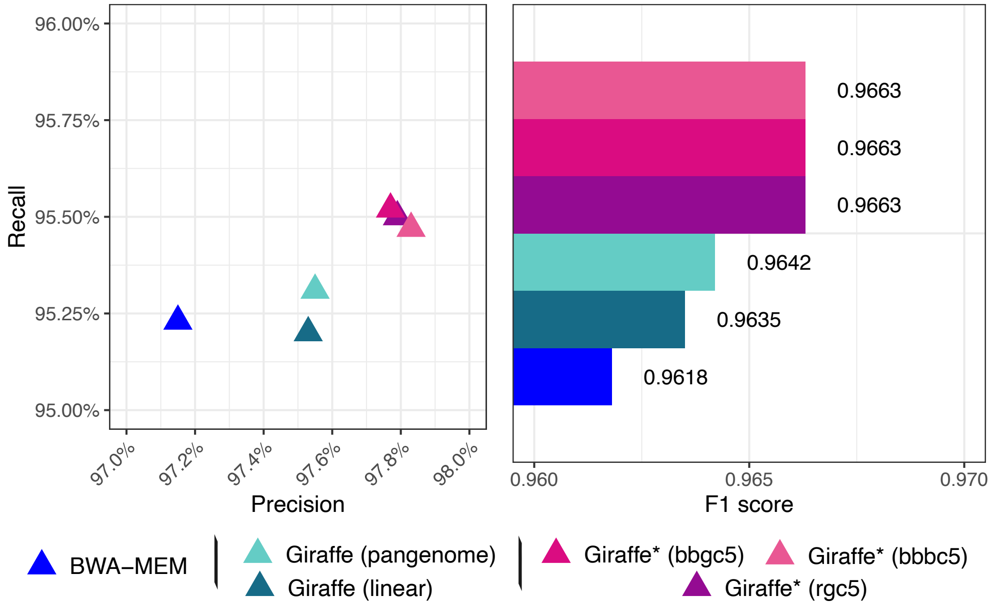

(a) Precision-recall measures & F1 scores for overall variants. "Giraffe" indicates use of the standard VG Giraffe aligner. "Giraffe*" indicates use of VG Giraffe with a personalized reference produced by the personalization workflow mention in parentheses.

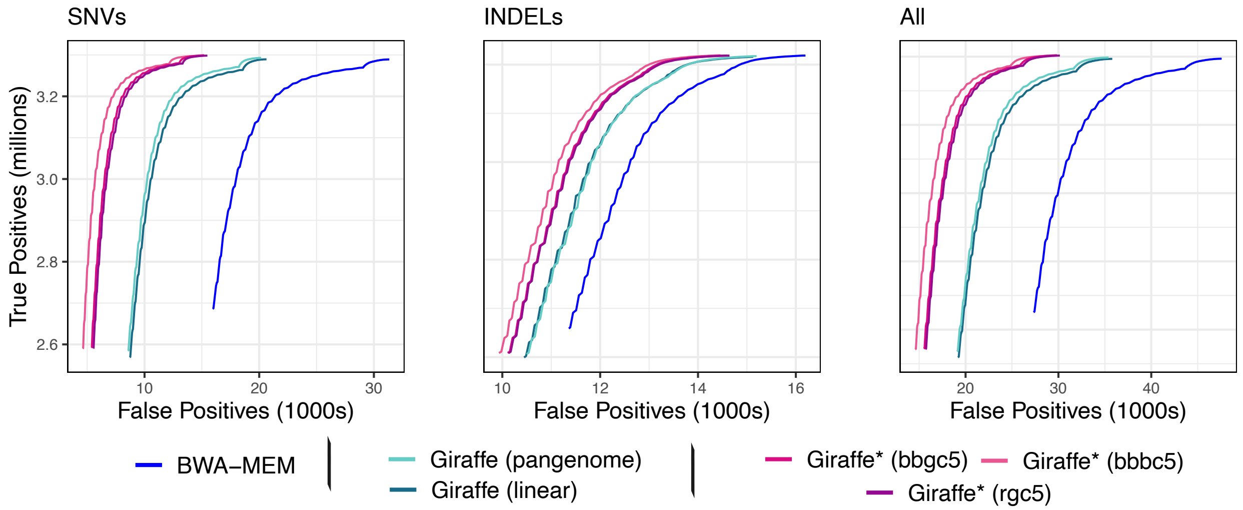

(b) ROC curves from `vcfeval` ROC data files for SNVs, indels and overall variants. ROC curves are stratified by variant quality value threshold. "Giraffe*" indicates use of VG Giraffe with a personalized reference produced by the personalization workflow mention in parentheses.

Figure S8: Variant calling accuracy metrics evaluated against T2TQ100 HG002 v1.0 truth VCF on GIAB HG002 high-confidence intersect regions. HG002 donor reads were aligned with a standard linear aligner (BWA-MEM) or one of various VG Giraffe workflows. "Giraffe" indicates use of the standard VG Giraffe aligner. "Giraffe*" indicates use of VG Giraffe with a personalized reference produced by the personalization workflow mention in parentheses.

bioRxiv preprint doi: https://doi.org/10.1101/2023.11.30.568362; this version posted May 16, 2024. The copyright holder for this preprint (which was not certified by peer review) is the author/funder, who has granted bioRxiv a license to display the preprint in perpetuity. It is made available under aCC-BY 4.0 International license.

Supplementary Tables

Table S1: Alignment score comparison between the `rgc1`-imputed diploid personalized reference and the standard linear GRCh38 reference.

**Alignment Score Difference (Personalized - GRCh38)**	**Read Count**	**Fraction (%)**	**Mean**	**Median**	**Variance**	**Std. Deviation**
Positive	50,065,960	96.71%	7.70	5.00	100.97	10.05
Negative	1,702,409	3.29%	-7.58	-5.00	112.81	10.62

bioRxiv preprint doi: https://doi.org/10.1101/2023.11.30.568362; this version posted May 16, 2024. The copyright holder for this preprint (which was not certified by peer review) is the author/funder, who has granted bioRxiv a license to display the preprint in perpetuity. It is made available under aCC-BY 4.0 International license.

Table S2: Variant calling performance metrics for HG001 real donor reads, stratified by SNVs, indels, and overall variants, across different reference combinations within GIAB HG001 high-confidence regions.

Mode	Workflow	Precision (%)	Recall (%)	F1 score
All	BWA-MEM	98.77	99.28	0.9902
	VG Giraffe (linear)	99.17	99.24	0.9921
	VG Giraffe (pangenome)	99.18	99.37	0.9928
	VG Giraffe* (rgc1)	99.33	99.54	0.9943
	VG Giraffe* (rgc5)	99.34	99.54	0.9944
	VG Giraffe* (bbgc5)	99.34	99.56	0.9945
	VG Giraffe* (bbbc5)	99.36	99.54	0.9945
SNV	BWA-MEM	98.77	99.29	0.9903
	VG Giraffe (linear)	99.21	99.28	0.9925
	VG Giraffe (pangenome)	99.22	99.41	0.9932
	VG Giraffe* (rgc1)	99.37	99.60	0.9949
	VG Giraffe* (rgc5)	99.38	99.60	0.9949
	VG Giraffe* (bbgc5)	99.38	99.63	0.9951
	VG Giraffe* (bbbc5)	99.41	99.60	0.9950
Indel	BWA-MEM	98.92	98.95	0.9894
	VG Giraffe (linear)	99.06	98.80	0.9893
	VG Giraffe (pangenome)	99.06	98.90	0.9898
	VG Giraffe* (rgc1)	99.18	98.96	0.9907
	VG Giraffe* (rgc5)	99.16	98.99	0.9908
	VG Giraffe* (bbgc5)	99.18	98.97	0.9908
	VG Giraffe* (bbbc5)	99.18	99.02	0.9910

Table S3: Variant calling performance metrics for HG001 real donor reads for overall variants, across different reference combinations within GIAB GRCh38 Complex Medically Relevant Gene (CMRG) regions.

Workflow	Precision (%)	Recall (%)	F1 score
BWA-MEM	85.37	97.87	0.9120
VG Giraffe (linear)	85.57	97.81	0.9128
VG Giraffe (pangenome)	85.58	98.03	0.9138
VG Giraffe* (rgc1)	85.55	98.22	0.9144
VG Giraffe* (rgc5)	85.57	98.21	0.9146
VG Giraffe* (bbgc5)	85.60	98.24	0.9148
VG Giraffe* (bbbc5)	85.77	98.19	0.9156

bioRxiv preprint doi: https://doi.org/10.1101/2023.11.30.568362; this version posted May 16, 2024. The copyright holder for this preprint (which was not certified by peer review) is the author/funder, who has granted bioRxiv a license to display the preprint in perpetuity. It is made available under aCC-BY 4.0 International license.

Table S4: Variant calling performance metrics for HG001 real donor reads for overall variants, across different reference combinations within GIAB GRCh38 stratifications.

Region	Workflow	Precision (%)	Recall (%)	F1 score
MHC	BWA-MEM	81.13	92.52	0.8646
	VG Giraffe (linear)	83.13	92.28	0.8746
	VG Giraffe (pangenome)	82.03	93.02	0.8718
	VG Giraffe* (rgc1)	84.63	92.59	0.8843
	VG Giraffe* (rgc5)	84.90	92.58	0.8858
	VG Giraffe* (bbgc5)	84.72	92.99	0.8867
	VG Giraffe* (bbbc5)	88.77	91.80	0.9026
alldifficultregions	BWA-MEM	56.08	91.45	0.6952
	VG Giraffe (linear)	59.21	91.72	0.7196
	VG Giraffe (pangenome)	59.39	92.13	0.7222
	VG Giraffe* (rgc1)	59.99	93.15	0.7298
	VG Giraffe* (rgc5)	60.04	93.13	0.7301
	VG Giraffe* (bbgc5)	60.00	93.28	0.7303
	VG Giraffe* (bbbc5)	60.25	93.17	0.7318
allOtherDifficultregions	BWA-MEM	18.08	82.75	0.2967
	VG Giraffe (linear)	23.28	82.59	0.3633
	VG Giraffe (pangenome)	23.20	84.37	0.3639
	VG Giraffe* (rgc1)	24.65	85.32	0.3824
	VG Giraffe* (rgc5)	24.71	85.28	0.3832
	VG Giraffe* (bbgc5)	24.70	85.62	0.3834
	VG Giraffe* (bbbc5)	24.98	84.25	0.3854
alllowmapandsegdupregions	BWA-MEM	40.27	81.74	0.5395
	VG Giraffe (linear)	47.10	83.40	0.6020
	VG Giraffe (pangenome)	48.27	84.21	0.6137
	VG Giraffe* (rgc1)	49.98	88.00	0.6375
	VG Giraffe* (rgc5)	50.06	87.90	0.6379
	VG Giraffe* (bbgc5)	50.03	88.46	0.6391
	VG Giraffe* (bbbc5)	50.33	88.26	0.6410

bioRxiv preprint doi: https://doi.org/10.1101/2023.11.30.568362; this version posted May 16, 2024. The copyright holder for this preprint (which was not certified by peer review) is the author/funder, who has granted bioRxiv a license to display the preprint in perpetuity. It is made available under aCC-BY 4.0 International license.

Table S5: Time and memory taken by each VG Giraffe-based workflow in the personalization, downstream indexing, and downstream alignment steps.

Phase	Workflow	Time (min)	Memory (GB)
Personalization	Impute-first: rgc1	50.23	14.45
	Impute-first: rgc5	94.97	14.45
	Impute-first: bbgc5	298.36	3.44
	Impute-first: bbbc5	329.17	34.78
Giraffe autoindex	linear	76.735	84.366
	pangenome	1817.522	397.303
	Impute-first: rgc1	86.483	113.902
	Impute-first: rgc5	91.604	114.077
	Impute-first: bbgc5	91.910	114.783
	Impute-first: bbbc5	87.335	115.220
Giraffe alignment	linear	137.529	53.062
	pangenome	204.824	70.086
	Impute-first: rgc1	157.973	65.249
	Impute-first: rgc5	158.795	63.799
	Impute-first: bbgc5	167.117	65.432
	Impute-first: bbbc5	160.250	65.440

Table S6: Index and graph size measurements for the VG Giraffe graphs generated in various workflows.

Input to `autoindex`	`.gbz` file (GB)	`.dist` file (GB)	`min` file (GB)	Total (GB)	Size (nodes + edges)
Linear GRCh38	2.6	1.5	35	39.1	193,945,149
1000 Genomes phase-3 pangenome	5.4	4.8	35	45.2	469,910,808
rgc1-imputed diploid	3.1	2.3	35	40.4	255,676,786
rgc5-imputed diploid	3.1	2.3	35	40.4	255,645,758
bbgc5-imputed diploid	3.1	2.3	35	40.4	256,688,029
bbbc5-imputed diploid	3.1	2.3	35	40.4	258,286,403

bioRxiv preprint doi: https://doi.org/10.1101/2023.11.30.568362; this version posted May 16, 2024. The copyright holder for this preprint (which was not certified by peer review) is the author/funder, who has granted bioRxiv a license to display the preprint in perpetuity. It is made available under aCC-BY 4.0 International license.

Table S7: Variant calling performance metrics for all variants present in HG001 across different minor allele frequency (MAF) ranges.

Variant Frequency	Workflow	Precision (%)	Recall (%)	F1 score
MAF ≥ 5% (n = 1,164,564)	BWA-MEM	99.77	99.67	0.9972
	VG Giraffe (linear)	99.81	99.69	0.9975
	VG Giraffe (pangenome)	99.84	99.78	0.9981
	VG Giraffe* (rgc1)	99.86	99.78	0.9982
	VG Giraffe* (rgc5)	99.86	99.78	0.9982
	VG Giraffe* (bbgc5)	99.86	99.79	0.9982
	VG Giraffe* (bbbc5)	99.86	99.78	0.9982
0.5% ≤ MAF < 5% (n = 171,987)	BWA-MEM	99.96	99.87	0.9992
	VG Giraffe (linear)	99.96	99.89	0.9993
	VG Giraffe (pangenome)	99.97	99.92	0.9994
	VG Giraffe* (rgc1)	99.97	99.92	0.9995
	VG Giraffe* (rgc5)	99.97	99.93	0.9995
	VG Giraffe* (bbgc5)	99.97	99.93	0.9995
	VG Giraffe* (bbbc5)	99.97	99.92	0.9995
MAF < 0.5% (n = 58,503)	BWA-MEM	99.98	99.71	0.9984
	VG Giraffe (linear)	99.99	99.76	0.9988
	VG Giraffe (pangenome)	99.99	99.78	0.9988
	VG Giraffe* (rgc1)	99.99	99.84	0.9992
	VG Giraffe* (rgc5)	99.99	99.85	0.9992
	VG Giraffe* (bbgc5)	99.99	99.83	0.9991
	VG Giraffe* (bbbc5)	99.99	99.83	0.9991

bioRxiv preprint doi: https://doi.org/10.1101/2023.11.30.568362; this version posted May 16, 2024. The copyright holder for this preprint (which was not certified by peer review) is the author/funder, who has granted bioRxiv a license to display the preprint in perpetuity. It is made available under aCC-BY 4.0 International license.

Table S8: Variant calling performance metrics for HG002 real donor reads, stratified by SNVs, indels, and overall variants, across different reference combinations using GIAB HG002 v4.2.1 truth within high-confidence regions.

Mode	Workflow	Precision (%)	Recall (%)	F1 score
All	BWA-MEM	98.77	99.17	0.9897
	VG Giraffe (linear)	99.24	99.09	0.9917
	VG Giraffe (pangenome)	99.24	99.22	0.9923
	VG Giraffe* (rgc5)	99.41	99.38	0.9939
	VG Giraffe* (bbgc5)	99.42	99.41	0.9941
	VG Giraffe* (bbbc5)	99.46	99.35	0.9941
SNV	BWA-MEM	98.77	99.20	0.9899
	VG Giraffe (linear)	99.28	99.14	0.9921
	VG Giraffe (pangenome)	99.27	99.27	0.9927
	VG Giraffe* (rgc5)	99.45	99.44	0.9945
	VG Giraffe* (bbgc5)	99.46	99.48	0.9947
	VG Giraffe* (bbbc5)	99.51	99.41	0.9946
Indel	BWA-MEM	98.89	98.85	0.9887
	VG Giraffe (linear)	99.11	98.71	0.9891
	VG Giraffe (pangenome)	99.12	98.76	0.9894
	VG Giraffe* (rgc5)	99.22	98.86	0.9904
	VG Giraffe* (bbgc5)	99.26	98.83	0.9905
	VG Giraffe* (bbbc5)	99.29	98.86	0.9908

bioRxiv preprint doi: https://doi.org/10.1101/2023.11.30.568362; this version posted May 16, 2024. The copyright holder for this preprint (which was not certified by peer review) is the author/funder, who has granted bioRxiv a license to display the preprint in perpetuity. It is made available under aCC-BY 4.0 International license.

Table S9: Variant calling performance metrics for HG002 real donor reads, stratified by SNVs, indels, and overall variants, across different reference combinations using T2TQ100 HG002 v1.0 truth within GIAB HG002 high-confidence regions.

Mode	Workflow	Precision (%)	Recall (%)	F1 score
All	BWA-MEM	98.77	98.68	0.9872
	VG Giraffe (linear)	99.07	98.67	0.9887
	VG Giraffe (pangenome)	99.08	98.77	0.9892
	VG Giraffe* (rgc5)	99.22	98.92	0.9907
	VG Giraffe* (bbgc5)	99.23	98.94	0.9908
	VG Giraffe* (bbbc5)	99.25	98.90	0.9907
SNV	BWA-MEM	99.07	99.29	0.9918
	VG Giraffe (linear)	99.39	99.29	0.9934
	VG Giraffe (pangenome)	99.40	99.40	0.9940
	VG Giraffe* (rgc5)	99.55	99.57	0.9956
	VG Giraffe* (bbgc5)	99.56	99.57	0.9957
	VG Giraffe* (bbbc5)	99.58	99.54	0.9956
Indel	BWA-MEM	96.92	94.93	0.9592
	VG Giraffe (linear)	97.11	94.79	0.9594
	VG Giraffe (pangenome)	97.12	94.84	0.9597
	VG Giraffe* (rgc5)	97.24	94.86	0.9604
	VG Giraffe* (bbgc5)	97.25	94.87	0.9605
	VG Giraffe* (bbbc5)	97.25	94.93	0.9608

bioRxiv preprint doi: https://doi.org/10.1101/2023.11.30.568362; this version posted May 16, 2024. The copyright holder for this preprint (which was not certified by peer review) is the author/funder, who has granted bioRxiv a license to display the preprint in perpetuity. It is made available under aCC-BY 4.0 International license.

Table S10: Variant calling performance metrics for HG002 real donor reads, stratified by SNVs, indels, and overall variants, across different reference combinations using T2TQ100 HG002 v1.0 truth within T2TQ100 HG002 v1.0 high-confidence regions.

Mode	Workflow	Precision (%)	Sensitivity (%)	F1 score
All	BWA-MEM	96.94	95.09	96.01
	VG Giraffe (linear)	97.39	95.03	96.19
	VG Giraffe (pangenome)	97.40	95.15	96.26
	VG Giraffe* (rgc5)	97.63	95.35	96.48
	VG Giraffe* (bbgc5)	97.61	95.38	96.48
	VG Giraffe* (bbbc5)	97.67	95.33	96.49
SNP	BWA-MEM	98.15	97.88	98.02
	VG Giraffe (linear)	98.64	97.90	98.27
	VG Giraffe (pangenome)	98.66	98.02	98.34
	VG Giraffe* (rgc5)	98.90	98.27	98.59
	VG Giraffe* (bbgc5)	98.90	98.27	98.59
	VG Giraffe* (bbbc5)	98.97	98.20	98.59
Indel	BWA-MEM	92.77	84.31	88.34
	VG Giraffe (linear)	92.99	83.99	88.26
	VG Giraffe (pangenome)	92.97	84.07	88.30
	VG Giraffe* (rgc5)	93.12	84.16	88.42
	VG Giraffe* (bbgc5)	93.27	84.04	88.42
	VG Giraffe* (bbbc5)	93.17	84.18	88.45

bioRxiv preprint doi: https://doi.org/10.1101/2023.11.30.568362; this version posted May 16, 2024. The copyright holder for this preprint (which was not certified by peer review) is the author/funder, who has granted bioRxiv a license to display the preprint in perpetuity. It is made available under aCC-BY 4.0 International license.

Table S11: Variant calling performance metrics for HG002 real donor reads for overall variants, across different reference combinations within GIAB GRCh38 Complex Medically Relevant Gene (CMRG) regions.

Workflow	Precision (%)	Recall (%)	F1 score
BWA-MEM	99.16	99.38	0.9927
VG Giraffe (linear)	99.39	99.33	0.9936
VG Giraffe (pangenome)	99.41	99.45	0.9943
VG Giraffe* (rgc5)	99.51	99.58	0.9954
VG Giraffe* (bbgc5)	99.48	99.59	0.9954
VG Giraffe* (bbbc5)	99.60	99.54	0.9957

bioRxiv preprint doi: https://doi.org/10.1101/2023.11.30.568362; this version posted May 16, 2024. The copyright holder for this preprint (which was not certified by peer review) is the author/funder, who has granted bioRxiv a license to display the preprint in perpetuity. It is made available under aCC-BY 4.0 International license.

Table S12: Variant calling performance metrics for HG002 real donor reads for overall variants, across different reference combinations within GIAB GRCh38 stratifications.

Region	Workflow	Precision (%)	Recall (%)	F1 score
MHC	BWA-MEM	97.81	97.46	0.9764
	VG_Giraffe(linear)	98.07	96.29	0.9717
	VG_Giraffe(pangenome)	99.25	98.05	0.9865
	VG_Giraffe*(rgc5)	99.46	98.19	0.9882
	VG_Giraffe*(bbgc5)	99.45	97.95	0.9869
	VG_Giraffe*(bbbc5)	98.67	94.06	0.9631
alldifficultregions	BWA-MEM	96.20	97.00	0.9660
	VG_Giraffe(linear)	97.85	96.69	0.9727
	VG_Giraffe(pangenome)	97.85	97.15	0.9750
	VG_Giraffe*(rgc5)	98.32	97.75	0.9803
	VG_Giraffe*(bbgc5)	98.35	97.87	0.9811
	VG_Giraffe*(bbbc5)	98.51	97.67	0.9809
allOtherDifficultregions	BWA-MEM	72.17	90.37	0.8025
	VG_Giraffe(linear)	83.83	89.58	0.8661
	VG_Giraffe(pangenome)	84.23	90.80	0.8739
	VG_Giraffe*(rgc5)	88.57	92.67	0.9057
	VG_Giraffe*(bbgc5)	88.82	92.95	0.9084
	VG_Giraffe*(bbbc5)	90.23	91.44	0.9083
alllowmapandsegdupregions	BWA-MEM	89.19	90.68	0.8993
	VG_Giraffe(linear)	94.51	90.01	0.9221
	VG_Giraffe(pangenome)	94.48	91.51	0.9297
	VG_Giraffe*(rgc5)	95.93	93.71	0.9481
	VG_Giraffe*(bbgc5)	96.08	94.18	0.9512
	VG_Giraffe*(bbbc5)	96.52	93.55	0.9501

bioRxiv preprint doi: https://doi.org/10.1101/2023.11.30.568362; this version posted May 16, 2024. The copyright holder for this preprint (which was not certified by peer review) is the author/funder, who has granted bioRxiv a license to display the preprint in perpetuity. It is made available under aCC-BY 4.0 International license.

Table S13: Variant calling performance metrics for HG002 real donor reads for overall variants, across different reference combinations within GIAB HG002 v1.0 Complex Medically Relevant Gene (CMRG) high-confidence regions.

Workflow	Precision (%)	Recall (%)	F1 score
BWA-MEM	94.70	95.10	0.9490
VG Giraffe (linear)	95.84	94.39	0.9511
VG Giraffe (pangenome)	95.83	95.05	0.9544
VG Giraffe* (rgc5)	93.57	95.35	0.9445
VG Giraffe* (bbgc5)	93.86	95.33	0.9459
VG Giraffe* (bbbc5)	95.25	95.23	0.9524

Table S14: Variant calling performance metrics for all variants present in HG002 across different minor allele frequency (MAF) ranges.

Variant Frequency	Workflow	Precision (%)	Recall (%)	F1 score
MAF $\geq$ 5% (n=1,179,717)	BWA-MEM	99.83	99.72	0.9977
	VG Giraffe (linear)	99.86	99.76	0.9981
	VG Giraffe (pangenome)	99.89	99.85	0.9987
	VG Giraffe* (rgc5)	99.90	99.84	0.9987
	VG Giraffe* (bbgc5)	99.90	99.85	0.9988
	VG Giraffe* (bbbc5)	99.90	99.80	0.9985
0.5% $\leq$ MAF $<$ 5% (n=175,103)	BWA-MEM	99.97	99.88	0.9992
	VG Giraffe (linear)	99.97	99.90	0.9994
	VG Giraffe (pangenome)	99.98	99.93	0.9995
	VG Giraffe* (rgc5)	99.98	99.93	0.9995
	VG Giraffe* (bbgc5)	99.98	99.94	0.9996
	VG Giraffe* (bbbc5)	99.99	99.90	0.9994
MAF $<$ 0.5% (n=60,273)	BWA-MEM	99.97	99.73	0.9985
	VG Giraffe (linear)	99.98	99.74	0.9986
	VG Giraffe (pangenome)	99.98	99.77	0.9988
	VG Giraffe* (rgc5)	99.99	99.83	0.9991
	VG Giraffe* (bbgc5)	99.98	99.85	0.9992
	VG Giraffe* (bbbc5)	99.98	99.84	0.9991

bioRxiv preprint doi: https://doi.org/10.1101/2023.11.30.568362; this version posted May 16, 2024. The copyright holder for this preprint (which was not certified by peer review) is the author/funder, who has granted bioRxiv a license to display the preprint in perpetuity. It is made available under aCC-BY 4.0 International license.

Table S15: Variant calling performance metrics for all variants present in HG002 CMRG v1.0 across different minor allele frequency (MAF) ranges.

Variant Frequency	Workflow	Precision (%)	Recall (%)	F1 score
MAF $\geq$ 5% (n=5,718)	BWA-MEM	99.54	98.51	0.9902
	VG Giraffe (linear)	99.60	98.24	0.9892
	VG Giraffe (pangenome)	99.58	98.53	0.9905
	VG Giraffe* (rgc5)	99.68	98.69	0.9918
	VG Giraffe* (bbgc5)	99.66	98.81	0.9923
	VG Giraffe* (bbbc5)	99.58	98.53	0.9905
0.5% $\leq$ MAF $<$ 5% (n=692)	BWA-MEM	99.68	99.21	0.9944
	VG Giraffe (linear)	99.84	99.05	0.9944
	VG Giraffe (pangenome)	99.84	99.21	0.9952
	VG Giraffe* (rgc5)	100.00	99.21	0.9960
	VG Giraffe* (bbgc5)	100.00	99.21	0.9960
	VG Giraffe* (bbbc5)	99.84	99.21	0.9952
MAF $<$ 0.5% (n=245)	BWA-MEM	98.88	92.67	0.9567
	VG Giraffe (linear)	98.88	93.19	0.9595
	VG Giraffe (pangenome)	98.88	93.19	0.9595
	VG Giraffe* (rgc5)	100.00	95.81	0.9786
	VG Giraffe* (bbgc5)	100.00	95.81	0.9786
	VG Giraffe* (bbbc5)	98.89	93.72	0.9623

Table S16: Summary of rare HG002 CMRG v1.0 variants recovered by *Impute-first (rgc5, bbgc5)* workflows in the TPO gene region [OMIM, gnomAD].

CHROM	POS	REF >ALT	dbSNP ID	gnomAD v4.1.0 ID[†]	Frequency[*]
chr2	1424939	C >T	rs113311329	2-1424939-C-T	0.001
chr2	1424955	T >C	rs541492938	2-1424955-T-C	0.0006
chr2	1424964	A >G	rs563945547	2-1424964-A-G	0.0014
chr2	1424965	A >C	rs531247861	2-1424965-A-C	0.0012

[*] dbSNP reported Variation Frequency (1000G).
[†] Rows 2& 3 are not observed in gnomAD records. All links show Variant Effect Predictor (VEP) annotation analysis. According to VEP annotation analysis, these variants fall on 10 transcripts in 2 genes suggesting a potential impact on gene function and possible clinical relevance.
