# Supplementary material for: Minimizing Reference Bias with an Impute-First Approach
Source: bioRxiv. 2023 Dec 2:2023.11.30.568362. Preprint. [Version 1] doi: 10.1101/2023.11.30.568362 (PMC10705441; doi:10.1101/2023.11.30.568362)
Supplement: Supplementary file 1 [file NIHPP2023.11.30.568362V1-supplement-1.pdf]

bioRxiv preprint doi: https://doi.org/10.1101/2023.11.30.568362; this version posted December 2, 2023. The copyright holder for this preprint (which was not certified by peer review) is the author/funder, who has granted bioRxiv a license to display the preprint in perpetuity. It is made available under aCC-BY 4.0 International license.

Supplementary Figures

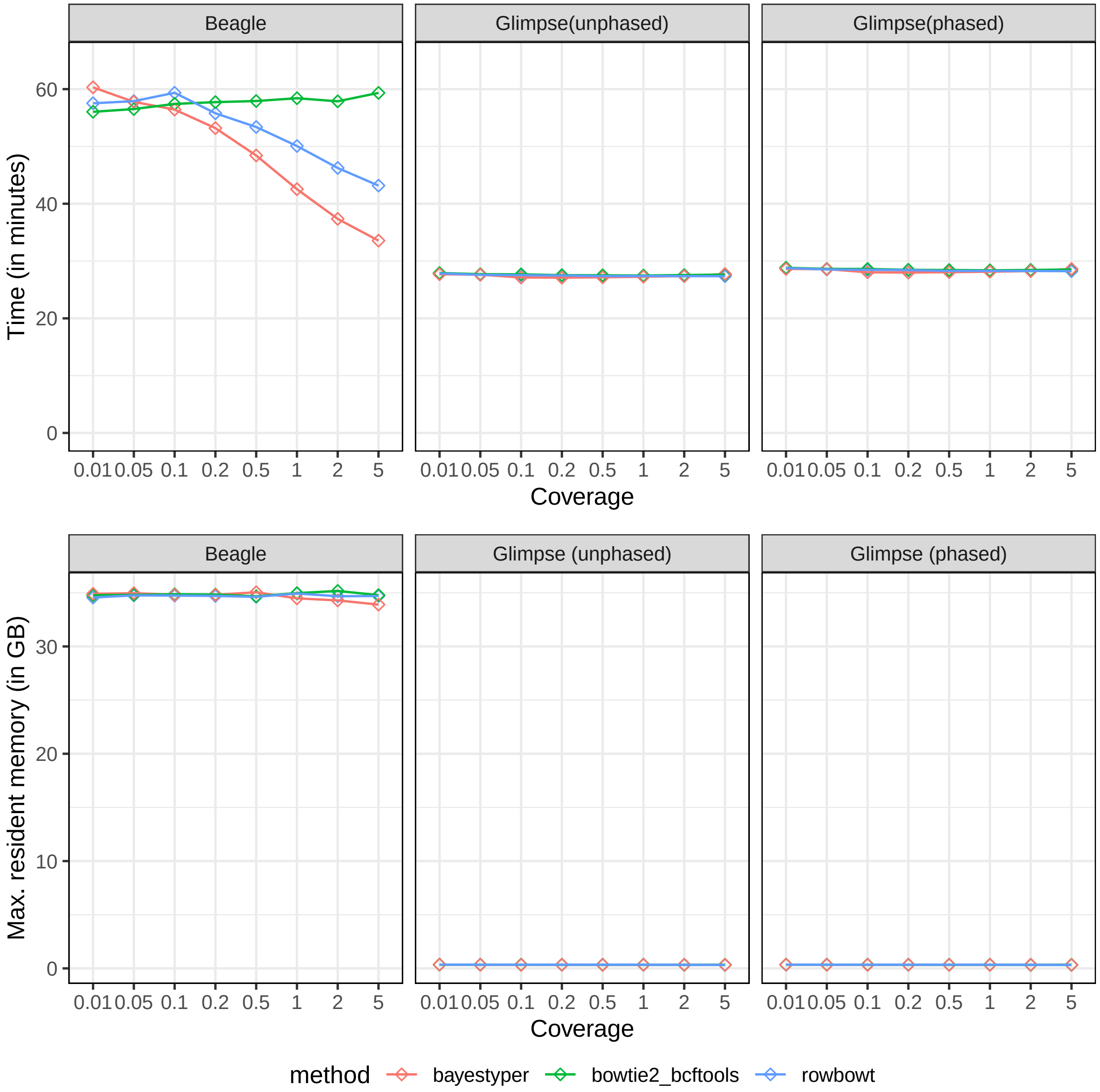

Figure S1: Computational overhead for Beagle and Glimpse stratified by the genotyping tool used upstraem, and as a function of the read coverage provided to the genotyper. Measurements for Glimpse were taken in both its phased and unphased modes.

bioRxiv preprint doi: https://doi.org/10.1101/2023.11.30.568362; this version posted December 2, 2023. The copyright holder for this preprint (which was not certified by peer review) is the author/funder, who has granted bioRxiv a license to display the preprint in perpetuity. It is made available under aCC-BY 4.0 International license.

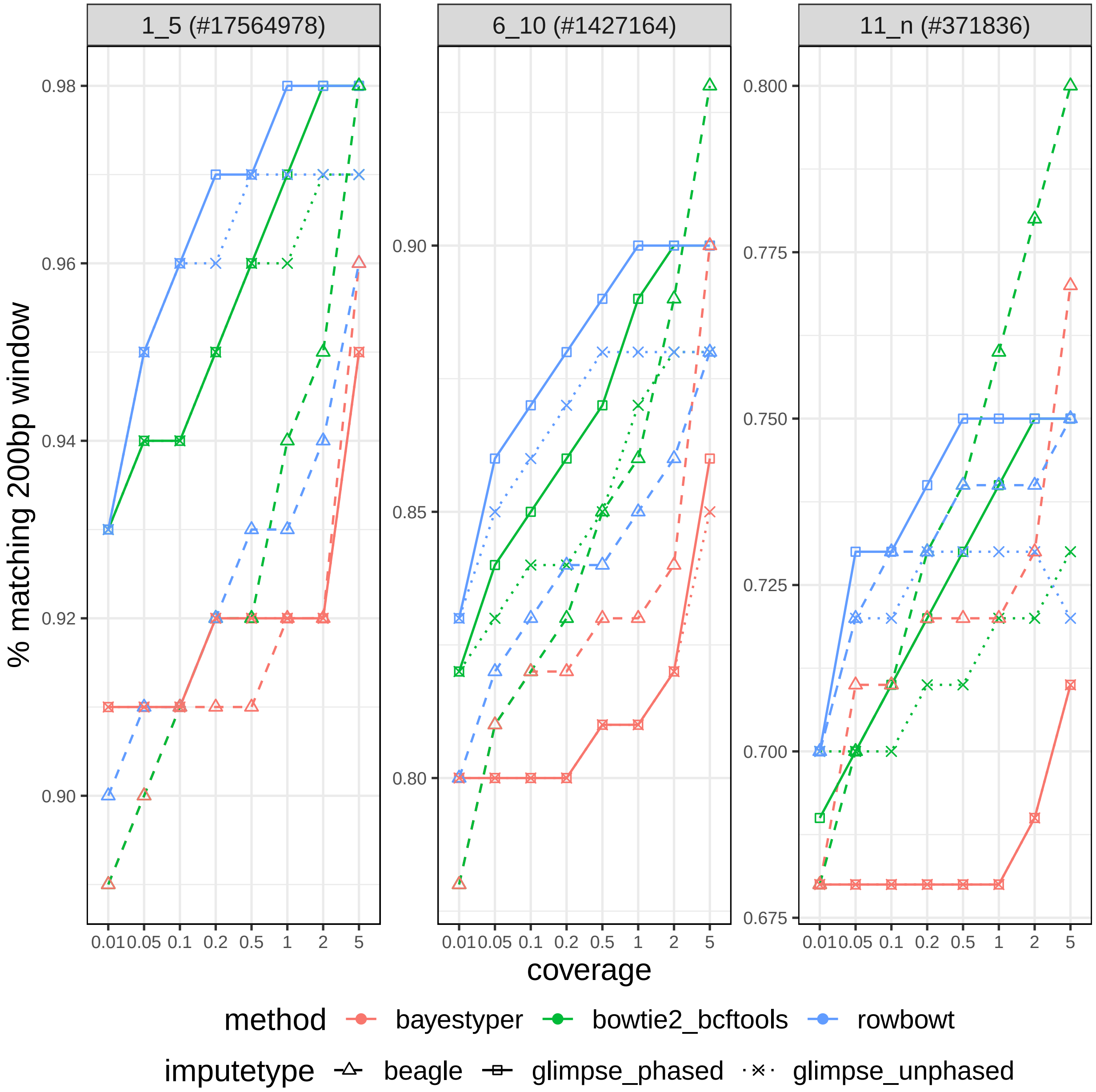

Figure S2: Window Accuracy for Beagle and Glimpse (in both phased and unphased modes) for different methods on diverse coverages.

bioRxiv preprint doi: https://doi.org/10.1101/2023.11.30.568362; this version posted December 2, 2023. The copyright holder for this preprint (which was not certified by peer review) is the author/funder, who has granted bioRxiv a license to display the preprint in perpetuity. It is made available under aCC-BY 4.0 International license.

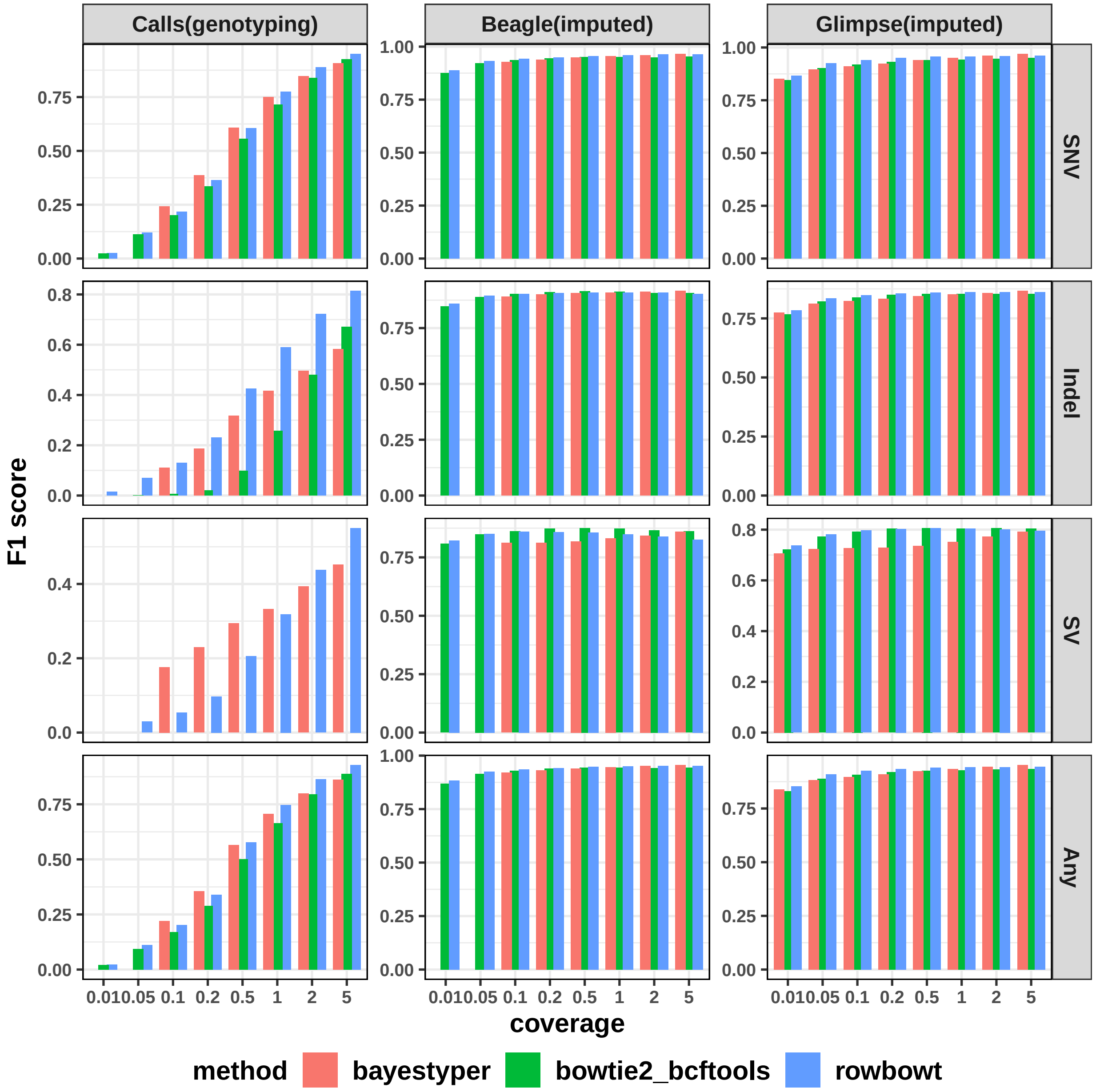

Figure S3: F1 score of alternate allele calls (ALT), stratified by variant type, for the personalized genomes constructed using each alignment/genotyping method in the Impute-first alignment workflow.

bioRxiv preprint doi: https://doi.org/10.1101/2023.11.30.568362; this version posted December 2, 2023. The copyright holder for this preprint (which was not certified by peer review) is the author/funder, who has granted bioRxiv a license to display the preprint in perpetuity. It is made available under aCC-BY 4.0 International license.

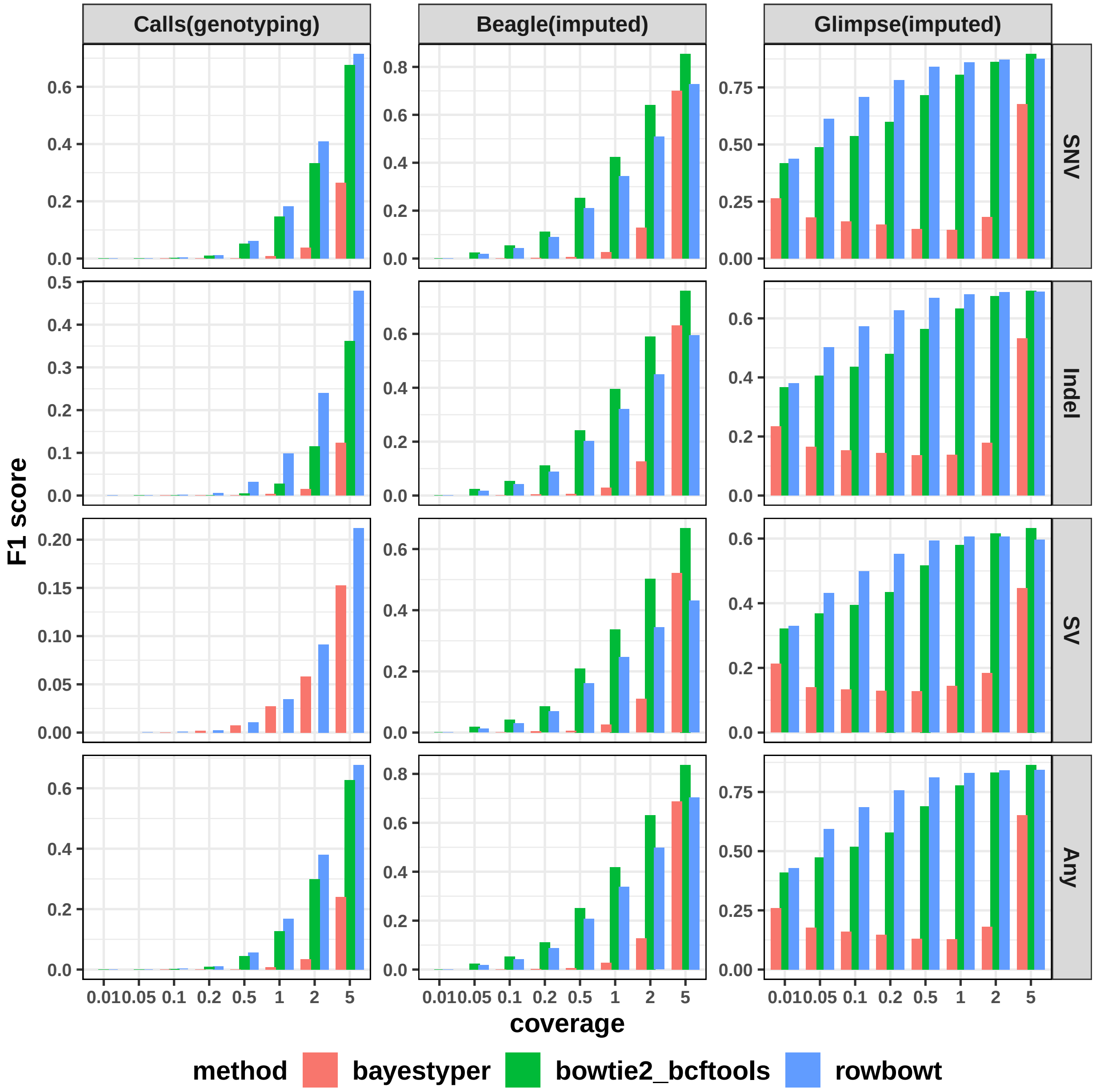

Figure S4: F1 score of heterozygous calls (HET), stratified by variant type, for the personalized genomes constructed using each alignment/genotyping method in the Impute-first alignment workflow.

bioRxiv preprint doi: https://doi.org/10.1101/2023.11.30.568362; this version posted December 2, 2023. The copyright holder for this preprint (which was not certified by peer review) is the author/funder, who has granted bioRxiv a license to display the preprint in perpetuity. It is made available under aCC-BY 4.0 International license.

Supplementary Tables

Table S1: Alignment score comparison between the rgc1-imputed diploid personalized reference and the standard linear GRCh38 reference.

Alignment Score Difference (Personalized - GRCh38)	Read Count	Fraction (%)	Mean	Median	Variance	Std. Deviation
Positive	50,065,960	96.71%	7.70	5.00	100.97	10.05
Negative	1,702,409	3.29%	-7.58	-5.00	112.81	10.62

bioRxiv preprint doi: https://doi.org/10.1101/2023.11.30.568362; this version posted December 2, 2023. The copyright holder for this preprint (which was not certified by peer review) is the author/funder, who has granted bioRxiv a license to display the preprint in perpetuity. It is made available under aCC-BY 4.0 International license.

Table S2: Variant calling performance metrics for HG001 real donor reads, stratified by SNVs, indels, and overall variants, across different reference combinations within GIAB HG001 high-confidence regions.

Mode	Workflow	Precision (%)	Recall (%)	F1 score
All	BWA-MEM	98.77	99.28	0.9902
	VG Giraffe (linear)	99.17	99.24	0.9921
	VG Giraffe (pangenome)	99.18	99.37	0.9928
	VG Giraffe* (rgc1)	99.33	99.54	0.9943
	VG Giraffe* (rgc5)	99.34	99.54	0.9944
	VG Giraffe* (bbgc5)	99.34	99.56	0.9945
	VG Giraffe* (bbbc5)	99.36	99.54	0.9945
SNV	BWA-MEM	98.77	99.29	0.9903
	VG Giraffe (linear)	99.21	99.28	0.9925
	VG Giraffe (pangenome)	99.22	99.41	0.9932
	VG Giraffe* (rgc1)	99.37	99.60	0.9949
	VG Giraffe* (rgc5)	99.38	99.60	0.9949
	VG Giraffe* (bbgc5)	99.38	99.63	0.9951
	VG Giraffe* (bbbc5)	99.41	99.60	0.9950
Indel	BWA-MEM	98.92	98.95	0.9894
	VG Giraffe (linear)	99.06	98.80	0.9893
	VG Giraffe (pangenome)	99.06	98.90	0.9898
	VG Giraffe* (rgc1)	99.18	98.96	0.9907
	VG Giraffe* (rgc5)	99.16	98.99	0.9908
	VG Giraffe* (bbgc5)	99.18	98.97	0.9908
	VG Giraffe* (bbbc5)	99.18	99.02	0.9910

bioRxiv preprint doi: https://doi.org/10.1101/2023.11.30.568362; this version posted December 2, 2023. The copyright holder for this preprint (which was not certified by peer review) is the author/funder, who has granted bioRxiv a license to display the preprint in perpetuity. It is made available under aCC-BY 4.0 International license.

Table S3: Variant calling performance metrics for HG001 real donor reads for overall variants, across different reference combinations within GIAB GRCh38 Complex Medically Relevant Gene (CMRG) regions.

Workflow	Precision (%)	Recall (%)	F1 score
BWA-MEM	85.37	97.87	0.9120
VG Giraffe (linear)	85.57	97.81	0.9128
VG Giraffe (pangenome)	85.58	98.03	0.9138
VG Giraffe* (rgc1)	85.55	98.22	0.9144
VG Giraffe* (rgc5)	85.57	98.21	0.9146
VG Giraffe* (bbgc5)	85.60	98.24	0.9148
VG Giraffe* (bbbc5)	85.77	98.19	0.9156

bioRxiv preprint doi: https://doi.org/10.1101/2023.11.30.568362; this version posted December 2, 2023. The copyright holder for this preprint (which was not certified by peer review) is the author/funder, who has granted bioRxiv a license to display the preprint in perpetuity. It is made available under aCC-BY 4.0 International license.

Table S4: Variant calling performance metrics for HG001 real donor reads for overall variants, across different reference combinations within GIAB GRCh38 stratifications.

Region	Workflow	Precision (%)	Recall (%)	F1 score
MHC	BWA-MEM	81.13	92.52	0.8646
	VG Giraffe (linear)	83.13	92.28	0.8746
	VG Giraffe (pangenome)	82.03	93.02	0.8718
	VG Giraffe* (rgc1)	84.63	92.59	0.8843
	VG Giraffe* (rgc5)	84.90	92.58	0.8858
	VG Giraffe* (bbgc5)	84.72	92.99	0.8867
	VG Giraffe* (bbbc5)	88.77	91.80	0.9026
alldifficultregions	BWA-MEM	56.08	91.45	0.6952
	VG Giraffe (linear)	59.21	91.72	0.7196
	VG Giraffe (pangenome)	59.39	92.13	0.7222
	VG Giraffe* (rgc1)	59.99	93.15	0.7298
	VG Giraffe* (rgc5)	60.04	93.13	0.7301
	VG Giraffe* (bbgc5)	60.00	93.28	0.7303
	VG Giraffe* (bbbc5)	60.25	93.17	0.7318
allOtherDifficultregions	BWA-MEM	18.08	82.75	0.2967
	VG Giraffe (linear)	23.28	82.59	0.3633
	VG Giraffe (pangenome)	23.20	84.37	0.3639
	VG Giraffe* (rgc1)	24.65	85.32	0.3824
	VG Giraffe* (rgc5)	24.71	85.28	0.3832
	VG Giraffe* (bbgc5)	24.70	85.62	0.3834
	VG Giraffe* (bbbc5)	24.98	84.25	0.3854
alllowmapandsegdupregions	BWA-MEM	40.27	81.74	0.5395
	VG Giraffe (linear)	47.10	83.40	0.6020
	VG Giraffe (pangenome)	48.27	84.21	0.6137
	VG Giraffe* (rgc1)	49.98	88.00	0.6375
	VG Giraffe* (rgc5)	50.06	87.90	0.6379
	VG Giraffe* (bbgc5)	50.03	88.46	0.6391
	VG Giraffe* (bbbc5)	50.33	88.26	0.6410

bioRxiv preprint doi: https://doi.org/10.1101/2023.11.30.568362; this version posted December 2, 2023. The copyright holder for this preprint (which was not certified by peer review) is the author/funder, who has granted bioRxiv a license to display the preprint in perpetuity. It is made available under aCC-BY 4.0 International license.

Table S5: Time and memory taken by each VG Giraffe-based workflow in the personalization, downstream indexing, and downstream alignment steps.

Phase	Workflow	Time (min)	Memory (GB)
Personalization	Impute-first: rgc1	50.23	14.45
	Impute-first: rgc5	94.97	14.45
	Impute-first: bbgc5	298.36	3.44
	Impute-first: bbbc5	329.17	34.78
Giraffe autoindex	linear	76.735	84.366
	pangenome	1817.522	397.303
	Impute-first: rgc1	86.483	113.902
	Impute-first: rgc5	91.604	114.077
	Impute-first: bbgc5	91.910	114.783
	Impute-first: bbbc5	87.335	115.220
Giraffe alignment	linear	137.529	53.062
	pangenome	204.824	70.086
	Impute-first: rgc1	157.973	65.249
	Impute-first: rgc5	158.795	63.799
	Impute-first: bbgc5	167.117	65.432
	Impute-first: bbbc5	160.250	65.440

Table S6: Index and graph size measurements for the VG Giraffe graphs generated in various workflows.

Input to `autoindex`	`.gbz` file (GB)	`.dist` file (GB)	`min` file (GB)	Total (GB)	Size (nodes + edges)
Linear GRCh38	2.6	1.5	35	39.1	193,945,149
1000 Genomes phase-3 pangenome	5.4	4.8	35	45.2	469,910,808
rgc1-imputed diploid	3.1	2.3	35	40.4	255,676,786
rgc5-imputed diploid	3.1	2.3	35	40.4	255,645,758
bbgc5-imputed diploid	3.1	2.3	35	40.4	256,688,029
bbbc5-imputed diploid	3.1	2.3	35	40.4	258,286,403

bioRxiv preprint doi: https://doi.org/10.1101/2023.11.30.568362; this version posted December 2, 2023. The copyright holder for this preprint (which was not certified by peer review) is the author/funder, who has granted bioRxiv a license to display the preprint in perpetuity. It is made available under aCC-BY 4.0 International license.

Variant Frequency	Workflow	Precision (%)	Recall (%)	F1 score
MAF $\geq$ 5% (n = 1,164,564)	BWA-MEM	99.77	99.67	0.9972
	VG Giraffe (linear)	99.81	99.69	0.9975
	VG Giraffe (pangenome)	99.84	99.78	0.9981
	VG Giraffe* (rgc1)	99.86	99.78	0.9982
	VG Giraffe* (rgc5)	99.86	99.78	0.9982
	VG Giraffe* (bbgc5)	99.86	99.79	0.9982
	VG Giraffe* (bbbc5)	99.86	99.78	0.9982
0.5% $\leq$ MAF $<$ 5% (n = 171,987)	BWA-MEM	99.96	99.87	0.9992
	VG Giraffe (linear)	99.96	99.89	0.9993
	VG Giraffe (pangenome)	99.97	99.92	0.9994
	VG Giraffe* (rgc1)	99.97	99.92	0.9995
	VG Giraffe* (rgc5)	99.97	99.93	0.9995
	VG Giraffe* (bbgc5)	99.97	99.93	0.9995
	VG Giraffe* (bbbc5)	99.97	99.92	0.9995
MAF $<$ 0.5% (n = 58,503)	BWA-MEM	99.98	99.71	0.9984
	VG Giraffe (linear)	99.99	99.76	0.9988
	VG Giraffe (pangenome)	99.99	99.78	0.9988
	VG Giraffe* (rgc1)	99.99	99.84	0.9992
	VG Giraffe* (rgc5)	99.99	99.85	0.9992
	VG Giraffe* (bbgc5)	99.99	99.83	0.9991
	VG Giraffe* (bbbc5)	99.99	99.83	0.9991

Table S7: Variant calling performance metrics for all variants present in HG001 across different minor allele frequency (MAF) ranges.
